# Xanthogranulomatous Salpingitis Associated with a Large Uterine Leiomyoma

**DOI:** 10.1155/2010/970805

**Published:** 2010-10-17

**Authors:** Joanne Margaret Howey, Etienne Mahe, Jasim Radhi

**Affiliations:** ^1^Michael G. DeGroote School of Medicine, McMaster University, 1280 Main Street West, Hamilton, ON, Canada L8S 4K1; ^2^Department of Pathology, McMaster University, 1280 Main Street West, Hamilton, ON, Canada L8S 4K1

## Abstract

A case of xanthogranulomatous salpingitis (XGS) associated with a large uterine leiomyoma in a 50-year-old woman is presented. Xanthogranulomatous inflammation is an uncommon form of chronic inflammation that is destructive to affected organs. It is characterized by the presence of lipid-filled macrophages with admixed lymphocytes, plasma cells, and neutrophils. A review of the literature revealed that most patients with XGS have a clinical history of long-standing pelvic inflammatory disease (PID) or, less often, endometriosis. We report a case lacking a history of either PID or endometriosis but with a concurrent large uterine leiomyoma. Although the exact etiology in this case was not clear, the leiomyoma may have played a contributory role in pathogenesis.

## 1. Introduction

Xanthogranulomatous inflammation is a form of chronic inflammation that is destructive to the normal tissue of the affected organs. It is characterized by the presence of a large number of lipid-containing macrophages with an admixture of lymphocytes, plasma cells, and neutrophils. Multinucleated giant cells may be present [1–4]. Multiple organs have been reported to be affected by xanthogranulomatous inflammation, most commonly the kidney followed by the gall bladder [5, 6]. Involvement of the female genital organs is rare, and only a few cases have been reported involving the fallopian tubes. Multiple etiologies have been postulated; chiefly, PID is the suspected culprit [7–10]. Other more general contributory factors in the pathogenesis of xanthogranulomatous inflammation may include necrosis, hemorrhage, and obstruction. Although pseudoxanthomatous salpingitis can be confused with XGS on histological examination, the two processes should be distinguished because of their different clinical associations [11].

## 2. Case Presentation

A 50-year-old, gravid 4, para 4, woman was evaluated for abnormal uterine bleeding. Her LMP was 6 months prior, and she was in otherwise good health. She had no history of endometriosis or PID and never used an IUD. She had a prior tonsillectomy and appendectomy, and her family history was unremarkable. She had a pelvic ultrasound that showed her uterus to be bulky and heterogeneous, containing a posterior uterine leiomyoma. Routine laboratory tests, including a *β*-HCG screen, were noncontributory.

Physical exam revealed an ectocervical polyp. The uterus was also markedly enlarged with a second-degree uterine prolapse as well as a cystocele and rectocele. Endocervical curettage was performed, and the polyp was sent for histology. The patient was advised to proceed with a total abdominal hysterectomy, possible bilateral salpingo-oophorectomy, and possible anterior and posterior enterocele repair. She was prescribed Marvelon (estrogen and progestin combination pill) to control her bleeding prior to the surgery. 

Total abdominal hysterectomy and left salpingo-oophorectomy were performed. The uterus was found to be bulky, globular, and mobile and enlarged to the size of a 14-week gestation. The left ovary was adherent to the left broad ligament posteriorly; at the time of surgery, this was thought to be due to inflammatory adhesions or endometriosis. On close examination, the left fallopian tube was swollen, resembling hydrosalpinx or hematosalpinx. The rest of the abdomen and pelvis were normal to inspection and palpation. The procedure was tolerated well, and the patient was discharged two days later.

## 3. Pathology

Gross examination of the specimen revealed a large, bulky uterus with serosal adhesions. On sectioning, the posterior myometrium showed a large 5.7 cm submucosal leiomyoma; the remainder of the uterus and cervix were unremarkable. The left fallopian tube was adherent to the ovary at its distal end and was distally enlarged to a caliber of 1.8 cm. Transection of the fallopian tube in this region revealed a thickened and fibrotic muscular wall with scanty hemorrhagic luminal contents. The ovary proper was unremarkable with the exception of a small follicular cyst.

**Figure 1:**
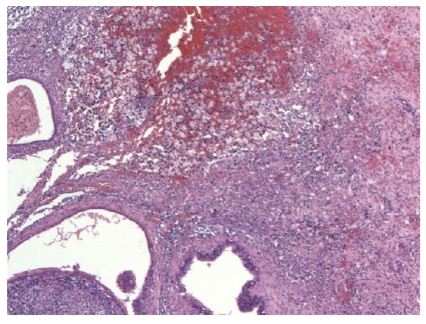
Fallopian tube showing marked inflammation and focal hemorrhage.

**Figure 2:**
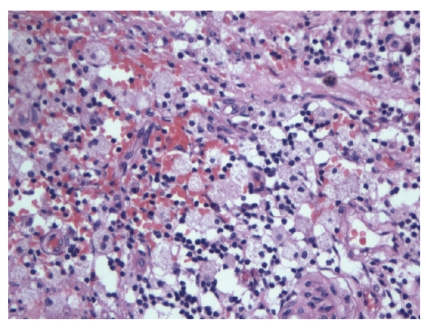
The inflammation is composed mainly of foamy histiocytes with scattered lymphocytes and plasma cells.

Histopathology of the cervical polyp proved it to be benign. The fallopian tubes showed infiltration of the lamina propria by large numbers of histiocyte with a mixture of inflammatory cells, including lymphocytes, plasma cells, and occasional neutrophils (Figure 1). There were also chronic inflammatory cells in the muscularis propria, and there were subserosal fibrosis and chronic inflammation with focal adhesions. The lumen contained numerous histiocytes and neutrophils. Many of the histiocytes in the lamina propria appeared to contain abundant lipid-like material (Figure 2). These findings were consistent with xanthogranulomatous salpingitis. Special stains for fungi and bacteria were negative.

## 4. Discussion

Xanthogranulomatous inflammation is characterized by the presence of numerous, foamy, lipid-rich histiocytes and other inflammatory cells [11]. This uncommon process is best known to occur in the kidney. Xanthogranulomatous inflammation has also been reported in the gallbladder, stomach, anorectal area, bone, urinary bladder, testis, epididymis, vagina, endometrium, ovary, and fallopian tube [9].

A review of the literature revealed fourteen cases of fallopian tube involvement reported to date [2, 9, 11–16]. Patients with XGS ranged from 23 to 72 years old. Initial presentation most often included lower abdominal or suprapubic pain, fever, menorrhagia, or vaginal bleeding, and physical examination typically showed a pelvic mass with adnexal tenderness [2, 14–19].

The exact pathogenesis of xanthogranulomatous inflammation remains debated. Many unrelated disorders may have the same mechanism of foam cell production; proposed causes are infection, ineffective antibiotic therapy, abnormality in lipid metabolism, endometriosis, and ineffective clearance of bacteria by phagocytes. Predisposing conditions for the development of XGS include PID, the presence of an IUD, endometriosis, and inborn error in lipid metabolism by macrophages, unsuccessful antibiotic therapy, and radiotherapy [7–10]. A combination of factors may also be suggested in some cases. For example, bleeding and obstruction may predispose to infection, and tissue necrosis occurs, followed by the release of cholesterol and other lipids and phagocytosis by macrophages.

XGS needs to be distinguished from PXS, also known as pseudoxanthomatous salpingiosis, which is an equally rare phenomenon and occurs mainly in women with a long history of endometriosis. PXS is characterized by numerous histiocytes containing brown lipofuscin (ceroid) pigment within the lamina propria. In our case, the lack of lipofuscin pigments and the presence of other inflammatory cells support the diagnosis of XGS.

Our patient presented with symptoms compatible with a submucosal leiomyoma. Lack of response to medical management of her menorrhagia led to a surgical hysterectomy, during which there was an operative suspicion of endometriosis because the left ovary was adherent to the left broad ligament. The patient did not have any history of a lipid metabolic disease, nor did her history give an indication of immunodeficiency. There was no evidence of endometriosis histologically; however, the fibrotic thickening with hemorrhage in the fallopian tube may be attributed to chronic inflammation. However, burnt out endometriosis can not be entirely excluded. Previous reports have suggested that xanthogranulomatous inflammation is probably initiated by a chronic obstructive and/or an ischemic process; this has been the experience of studies looking into xanthogranulomatous inflammation of the genital tract, kidney, and gall bladder [20]. It is possible, in this case, that the presence of a large leiomyoma and a history of prolapse may have led to some degree of obstruction of the fallopian tube and/or circulatory disturbance, possibly resulting in congestion, chronic ischemia, and predisposition to infection with subsequent development of XGS.
